# Supplementary material for: High-resolution computed tomography with scattered X-ray radiation and a single pixel detector
Source: Commun Eng. 2024 Mar 18;3:39. doi: 10.1038/s44172-024-00184-6 (PMC10955963; doi:10.1038/s44172-024-00184-6)
Supplement: Supplementary file 2 — Supplementary Information [file 44172_2024_184_MOESM2_ESM.pdf]

High-resolution computed tomography with scattered X-ray radiation and a single pixel detector

A. Ben Yehuda[1], O. Sefi[1], Y. Klein[1], H. Schwartz[1], E. Cohen[2], R. H Shukrun[1,3] and S. Shwartz[1*]

Supplementary Information

Supplementary Note 1 | Scanning procedure

The measurement time at each mask position was 5 seconds. We repeated measurements for 3468 different mask positions at each angle of the sample, totaling 28 object angles. The angle of the object was adjusted using a rotation stage after every mask scan.

Supplementary Note 2 | Spatial resolution

The spatial resolution of our method can be evaluated by measuring the width of the autocorrelation function of the mask that modulates the input X-ray beam [1]. The autocorrelation function of the mask we used in another, lower energy experiment is presented in Supplementary Figure 2(b), while the 1D horizontal and vertical projections are presented in Supplementary Figure 2(c) and Supplementary Figure 2(d), respectively. The autocorrelation function is nearly isotropic and the FWHM of the curve are 175 µm and 127 µm for the horizontal and vertical axes, respectively. We used this example because we did not have a high-resolution X-ray camera for high energy X-ray which would allow us to take images of the silver mask we used in the main experiment.

Another method for assessing spatial resolution involves the use of Fourier Ring Correlation (FRC). FRC determines resolution by cross-correlating two distinct images of the same object across different spatial frequencies, often visualized as rings in Fourier space. When noise or artifacts significantly affect the signal, it results in a low correlation, revealing constraints in measurement resolution.

The FRC plot for the main experiment is presented in Fig. 4l, and the analysis suggests that the resolution of the primary experiment is approximately 500 µm.

Supplementary Note 3 | Experiment at lower photon energy

To validate our method, we also performed an additional experiment with an x-ray tube that emits radiation at 20 keV.

Since we used sandpaper for the mask, we measured it without the object and registered the profiles of the intensity at the object plane by acquiring the images at the various positions of the mask. The experimental setup of this step is described in Supplementary Figure 3(a), In the second step, which is depicted in Supplementary Information Fig .3(b), we inserted the object, the figure 7 made from a polylactic acid (PLA) as shown in Supplementary Figure 3(c). We measured the x-ray scattering with two energy resolving silicon drift detectors (SDDs) located at two different positions as is shown in Supplementary Figure 3(b). We then raster scanned the sandpaper and recorded the radiation, which was scattered from the object.

Because the number of pixels in this experiment was only 31 by 40, we used the TVAL3 algorithm [2] for the image reconstruction to avoid the reduction in the resolution that is the results of the pixel resize required by the constraints of the GIDC code.
We reconstructed the image of the object by using the scattered radiation that was collected by the detectors for 3000 realizations as shown in Supplementary Figure 4. The number of pixels in the image is 1240 and at each realization we measured about $2\cdot10^6$ scattered photons from the object. Based on edge blurring, we estimate that the spatial resolution of our proof-of-principle experiment was about 150 µm. This agrees with the width of the measured autocorrelation function in Supplementary Figure 4 and indicates that further improvement of the spatial resolution is feasible by a simple reduction of the speckle size [3]. As shown in Supplementary Figure 4 each detector produced a slightly different image reconstruction, with areas containing more absorbing material appearing darker. This is due to self-absorption of the scattering object. For example, in Supplementary Figure 4(b) the detector is on the right side of the object, resulting in a darker upper section of the image, as more material was present in that area. However, the bottom of the image was less affected by this effect due to the smaller size of the material, which blocked less radiation. By taking the average of both images produced by each detector, we were able to produce a better, more homogeneous image, as we show in Supplementary Figure 4(c). The findings of the study highlight the critical role played by the position of the detector in relation to the object. This positioning can yield additional insights and should be considered when reconstructing images.

Supplementary Note 4 | Simulation results

To validate the results of the low energy experiment we performed a Monte Carlo (MC) simulation at high energy x-ray (80kVp). MC simulations consider the complex light-matter interactions and are able to solve the radiation transfer problem for the relevant geometry and initial conditions. By using such simulation tools, we provide a deeper insight into the physical mechanism of our experimental observation, an insight that would have been impossible to obtain via traditional simulations that consider only geometrical optics. The simulation results strengthen the findings of our experiment and prove that the positioning of the detector has a major effect on the reconstructed image (Supplementary Figure 5).

Supplementary Note 5 | Number of samplings

To quantify the quality of the reconstructed image we defined the “convergence factor” C_F:

$$C_F = \frac{1}{N\cdot M}\sum_{i,j=1}^{N,M}(x_{i,j} - b_{i,j})^2. \quad \textbf{(S1)}$$

Here $x_{i,j}$ is the i_{th} and j_{th} point in the reconstructed image, $b_{i,j}$ represents the best value for a reconstructed image using 6000 samplings and N and M represent the number of points in every dimension. In Supplementary Figure 6 we show the C_F as a function of the number of samplings. It is evident that beyond 3000 samplings the improvement of the reconstructed image becomes insignificant.

Figures and Tables

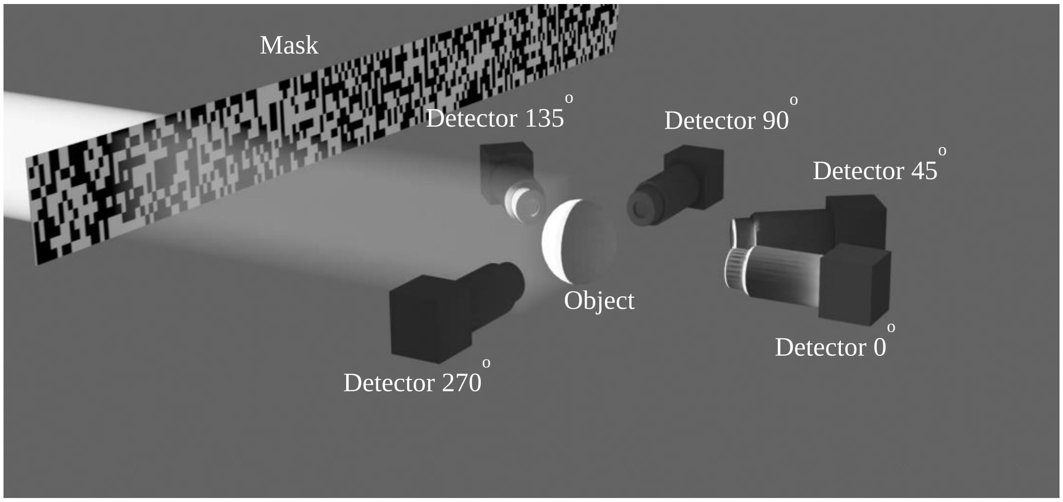

Supplementary Figure 1|Schematic illustration of the simulation setup. *The simulated mask modulates the beam, which irradiates the simulated object (ball shaped bone) with known random binary speckle patterns. The scattered x-ray radiation is then collected by single pixel detectors mounted at different angles. The resulting signals are then used to reconstruct a computational image from different angles.*

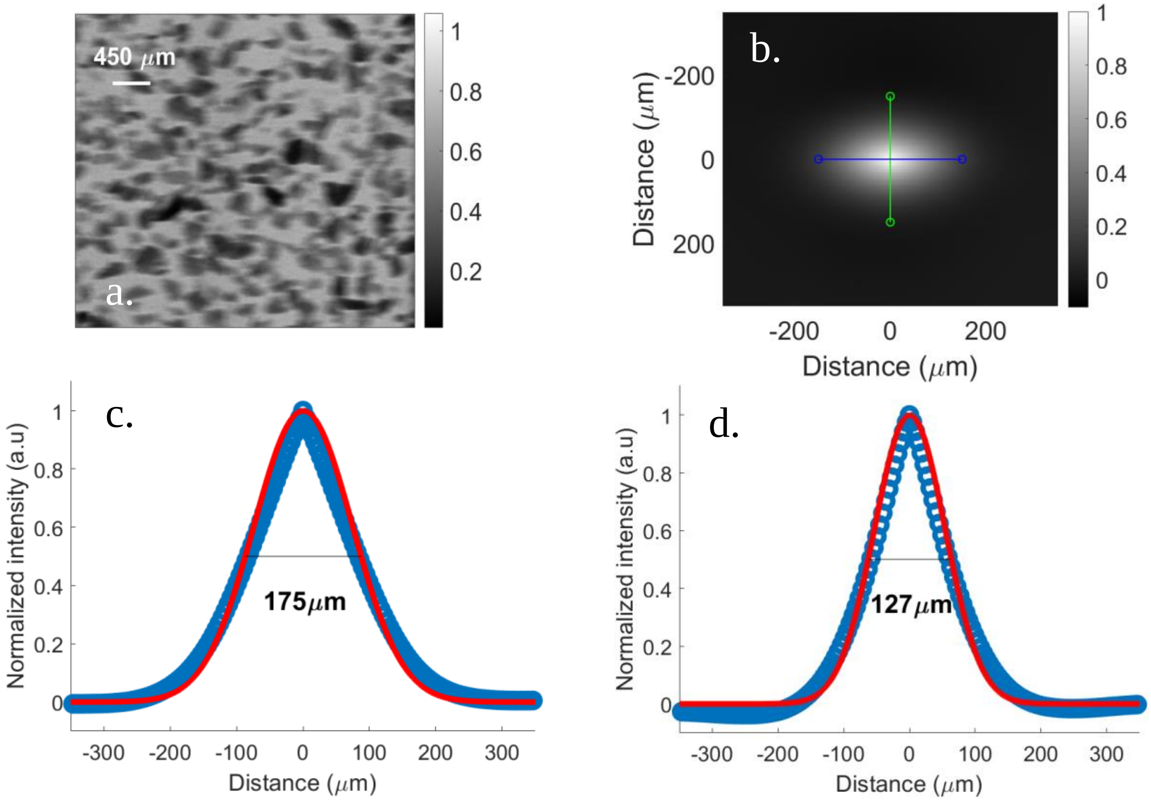

Supplementary Figure 2|Spatial resolution assessment. *(a) Example of the reference data (the intensity fluctuations induced by the mask). (b) Autocorrelation function of the intensity pattern induced by the mask. The blue and green lines indicate the horizontal and vertical cross sections shown in (c) and (d). The blue dots are the measured data for the horizontal and vertical cross sections and the red curves are interpolation functions.*

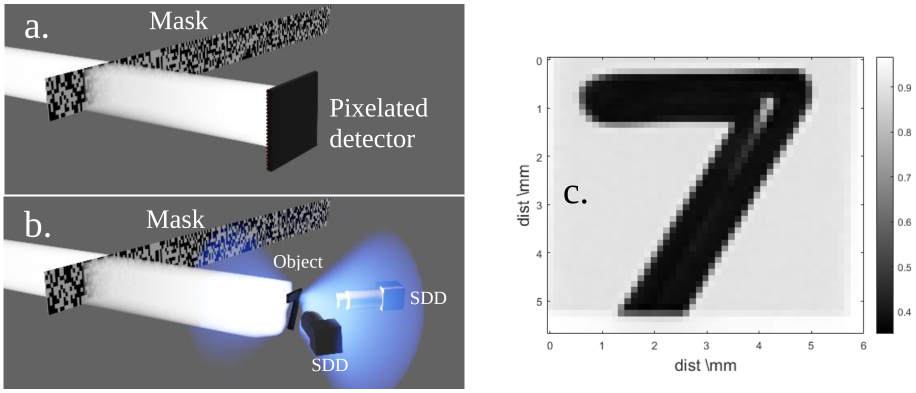

Supplementary Figure 3|Schematic of the experimental setup and the sample. *In step 1 (a), we measure the intensity patterns induced by the mask in the absence of the objects. In step 2 (b) we measure the radiation scattered off the object from both its sides. (c) The image obtained by direct imaging of the object.*

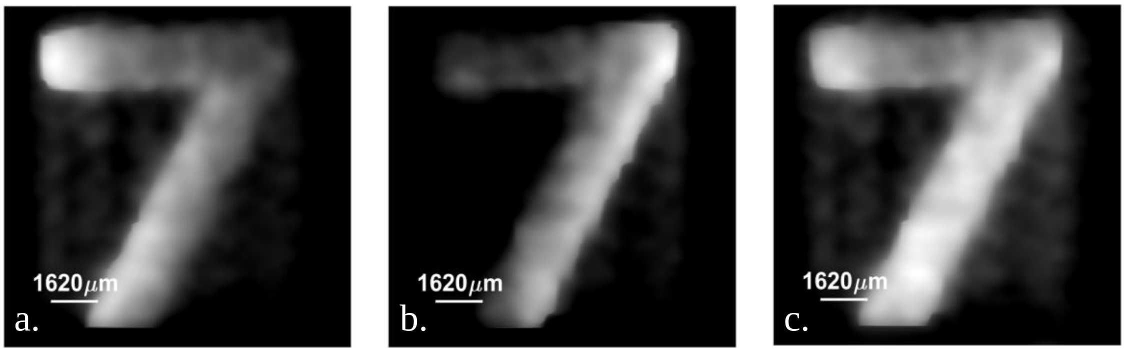

Supplementary Figure 4|Reconstruction images of the object with 3000 realizations. *(a) Reconstruction of the object which was sampled with the detector we positioned on the left side of the object. (b) Reconstruction of the object which was sampled with the detector we positioned on the right side of the object (c) Average of (a) and (b). This comparison shows that different detector positions result in different image reconstructions due to self-absorption and scatter of the object. These results provide valuable insights on the density and composition of the object.*

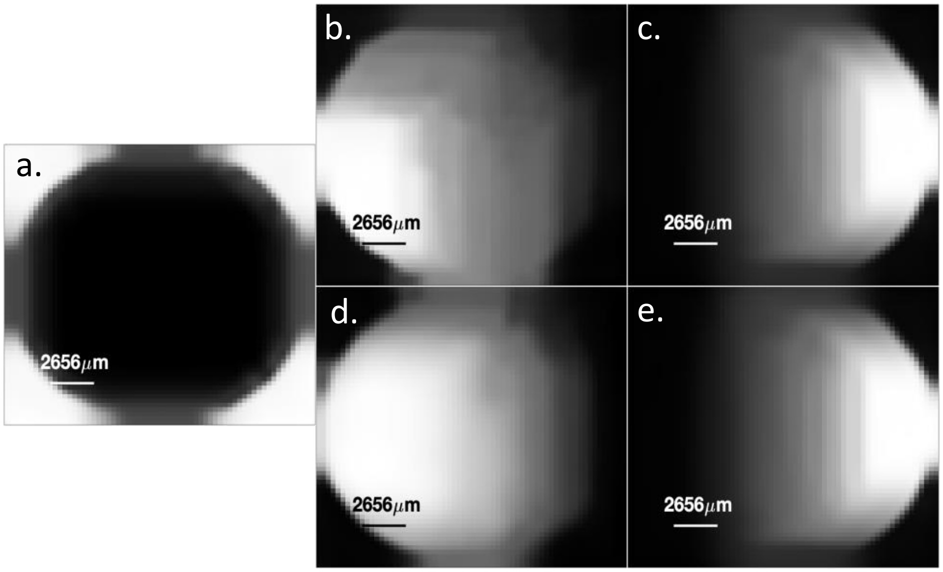

Supplementary Figure 5|Simulation results show the reconstructed images for the detector at*: (a) 0 degrees (transmission), (b) 45 degrees, (c) 90 degrees, (d) 135 degrees, and (e) 270 degrees. These simulation results qualitatively agree with our experimental results and support the claim that different detector positions result in different scatter image reconstructions due to physical effects such as self-absorption and self-scattering of the object.*

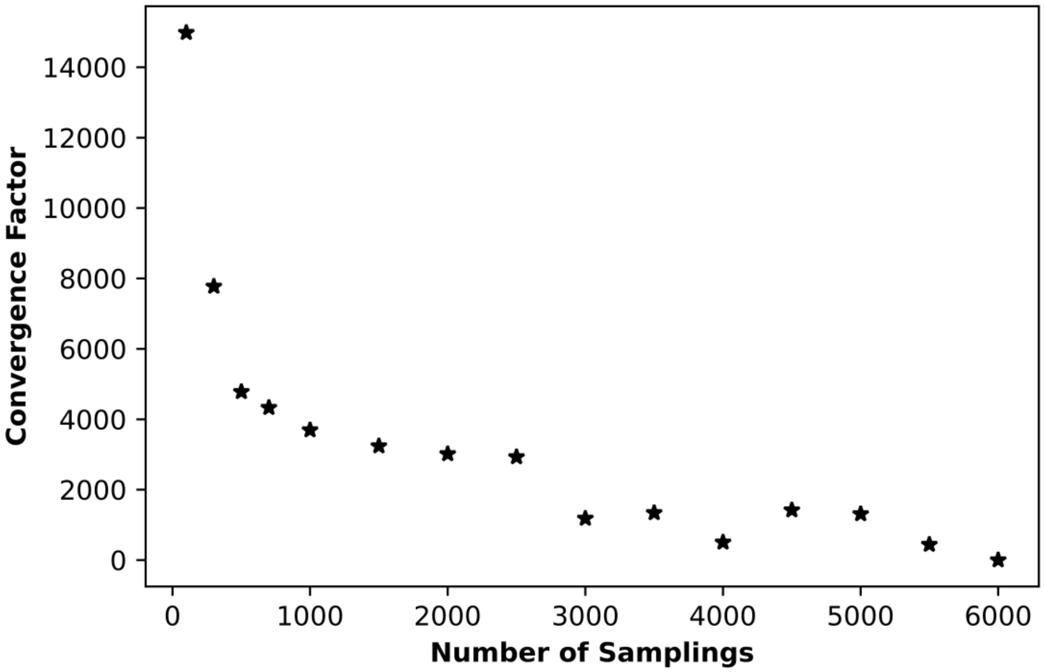

Supplementary Figure 6|Convergence Factor as a function of the number of samplings: The data were calculated according to Eq. S1. It is evident that beyond 3000 samplings the improvement of the reconstructed image becomes insignificant.

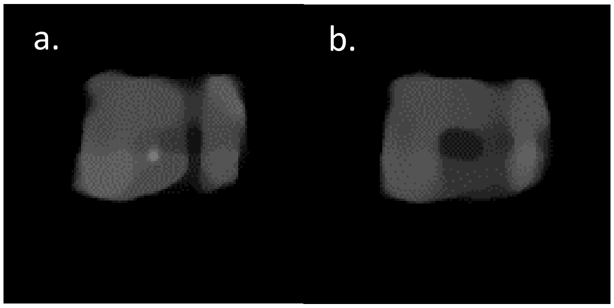

Supplementary Figure 7|Two tomogram reconstructions we used for the Fourier ring correlation analysis: Both tomograms were reconstructed from a selection of 3000 random different samplings out of a total of 3468.

```
img_W = 128
img_H = 128
SR = 0.211                                      # sampling rate
batch_size = 1
lr0 = 0.002                                     # learning rate
TV_strength = 1*10^-3.9                         # regularization parameter of Total Variation
num_patterns = int(np.round(img_W*img_H*SR))    # number of measurement times
Steps = 1501                                    # optimization steps
```

Supplementary Figure 8|GIDC algorithm code parameters (Python)

```
Angles=[0, 180, 90, 270, 45, 225, 315, 135, 22.5, 202.5, 67.5, 247.5, 112.5, 292.5, 157.5,
337.5, 355, 175, 348, 168, 353, 173, 5, 185, 12, 192, 300, 120];
niter=500;
angles=Angles*(pi/180)
geo=defaultGeometry('mode','parallel','nDetector',[128 128]);
imgSART_TV=SART_TV(projections,geo,angles,niter,'TVlambda',1000,'TViter',1000);
```

Supplementary Figure 9|Computational tomography reconstruction tool code parameters (MATLAB)

	**material**	**Density (g/cm3)**	**Elemental composition (% by mass)**
**Object**	Bone	1.85	H-4.7234, C-14.433, N-4.199, O-44.6096, Mg-0.22, P-10.497, S-0.0315, Ca-20.993
**Surrounding media**	Air	0.00120484	C-1.248, N-75.5267, O-23.1781, Ar-1.2827

Supplementary Table 1|Simulation geometry and materials

Supplementary References

1. Gatti, A., Brambilla, E., Bache, M. & Lugiato, L. A. Ghost imaging with thermal light: comparing entanglement and classical correlation. *Phys Rev Lett* **93**, (2004).
2. Li, C. *et al.* An efficient augmented Lagrangian method with applications to total variation minimization. *Comput Optim Appl* **56**, 507–530 (2013).
3. Klein, Y., Sefi, O., Schwartz, H. & Shwartz, S. Chemical element mapping by x-ray computational ghost fluorescence. *Optica* **9**, 63–70 (2022).
